# An* In Situ* and* In Silico* Evaluation of Biophysical Effects of 27 MHz Electromagnetic Whole Body Humans Exposure Expressed by the Limb Current

**DOI:** 10.1155/2017/5785482

**Published:** 2017-07-05

**Authors:** Jolanta Karpowicz, Patryk Zradziński, Jarosław Kieliszek, Krzysztof Gryz, Jaromir Sobiech, Wiesław Leszko

**Affiliations:** ^1^Central Institute for Labour Protection-National Research Institute, Laboratory of Electromagnetic Hazards, Warszawa, Poland; ^2^Military Institute of Hygiene and Epidemiology, Laboratory of Electromagnetic Radiation Metrology, Warszawa, Poland

## Abstract

**Objectives:**

The aim was to evaluate correlations between biophysical effects of 27 MHz electromagnetic field exposure in humans (limb induced current (LIC)) and (1) parameters of affecting heterogeneous electric field and (2) body anthropometric properties, in order to improve the evaluation of electromagnetic environmental hazards.

**Methods:**

Biophysical effects of exposure were studied* in situ* by measurements of LIC in 24 volunteers (at the ankle) standing near radio communication rod antenna and* in silico* in 4 numerical body phantoms exposed near a model of antenna.

**Results:**

Strong, positive, statistically significant correlations were found in all exposure scenarios between LIC and body volume index (body height multiplied by mass) (*r* > 0.7; *p* < 0.001). The most informative exposure parameters, with respect to the evaluation of electromagnetic hazards by measurements (i.e., the ones strongest correlated with LIC), were found to be the value of electric field (unperturbed field, in the absence of body) in front of the chest (50 cm from body axis) or the maximum value in space occupied by human. Such parameters were not analysed in previous studies.

**Conclusions:**

Exposed person's body volume and electric field strength in front of the chest determine LIC in studied exposure scenarios, but their wider applicability needs further studies.

## 1. Introduction

At present, wireless communication belongs to the basic tools used by various rescue and uniformed services, including military units [1–4]. Such devices include a portable radio communication units (known as a radiophones) equipped with rod antenna and operating at a frequency of approximately 27 MHz of emitted electromagnetic radiation (EMR), used by both military and civilian services. Devices of this type provide good quality connections at longer distances than public mobile phone systems and even in an environment not covered by public wireless communication systems, because they may be equipped with terminals of significantly higher output power than public mobile phone systems.

Human exposure to EMR was identified as an environmental factor that may create health and safety hazards, including a potentially carcinogenic outcome of exposure [5–8]. The environmental impact of EMR is characterized by the strength of electric and magnetic fields (*E*-field and *H*-field, resp.), representing its indivisible components [9]. European Directive 2013/35/EU sets out the minimum requirements regarding the protection of workers against health and safety hazards caused by electromagnetic fields (EMF) in the workplace [10]. According to it, as well as according to international guidelines provided by International Commission on Nonionizing Radiation Protection (ICNIRP) and standard from Institute of Electrical and Electronics Engineers (IEEE), in the radiofrequency band used in radio communication (including 27 MHz), the exposure limitations are provided to protect against the thermal effects caused by EMF biophysical influence [5, 11]. Such Exposure Limit Values (ELVs) are specified with regard to the values of the specific energy absorption rate (SAR). SAR is defined as the ratio between absorbed energy and unit weight of tissue (in W/kg) and is only assessable by numerical calculations (recognized as* in silico* measurements), requiring complex research procedures [5, 9–14]. SAR limits are provided for the effects of exposure averaged over a whole body or localized values as well. For this reason, various numerical phantoms of human body were developed, with different numerical parameters, internal organ details, and anthropometric properties, such as height and body mass [15, 16]. Results of evaluation of effects of EMF exposure in the human body, carried out by* in silico* measurements, are dependent on both the exposure conditions and structure of the phantoms [9, 17].

Directive 2013/35/EU, ICNIRP, and IEEE also accept a simplified assessment of the biophysical exposure effects and compliance with SAR limits, which can be done using* in situ* or* in silico* measurements of *E*-field strength (*E* in V/m), *H*-field strength (*H* in A/m), and limb induced current (*I*_L_ in mA), which all need to be lower than their relevant limits [5, 10, 11]. Limits set with respect to limb induced current (LIC) values play a practical role of measurable substitute for the localized SAR evaluation in limbs (achievable only by numerical measurements). Consequently, LIC measurements may be applicable in the evaluation of electromagnetic hazards in the real work environment, as well as validating numerical modelling in the research. Measurements of LIC do not characterize biophysical effects of electromagnetic exposure in other body parts.

The aim of the study was to evaluate correlations between the biophysical effects of EMR exposure in the human body, represented by LIC, and (1) the parameters of heterogeneous E-field affecting humans and (2) the anthropometric properties of the human body, with respect to improvement of the protocol of environmental electromagnetic hazards evaluation.

## 2. Material and Methods 

The investigations involved the following:

- Experimental studies (*in situ* measurements) into the LIC (at ankle) in volunteers staying close to EMR source and mapping the *E*-field spatial distribution near the source to create and validate the numerical model of this EMR source and the EMR exposure scenario to be used in* in silico* measurements
- A series of numerical calculations (*in silico* measurements) representing a whole body exposure to an EMR of 27 MHz, a scenario that mimics the one of* in situ* measurements (with respect to *E*-field spatial distribution), and also the biophysical influence of EMR on various human body numerical phantoms (with respect to LIC)
- Determining the correlation between the values of LIC (measured* in situ* or* in silico*) and (1) various parameters of spatially heterogeneous *E*-field characterizing exposure with respect to the EMR coupling with a body of individual properties and (2) the anthropometric properties of the human body (volunteers and numerical phantoms)
- Analysis of LIC in humans exposed to heterogeneous EMR from radiophone rod antenna of *E*-field strength equal to limits of exposure established by labour law in European Union (Directive 2013/35/EU, further mentioned as D2013/35/EU [10]).

### 2.1. Field Source: Rod Antenna of a Radiophone

**Figure 1:**
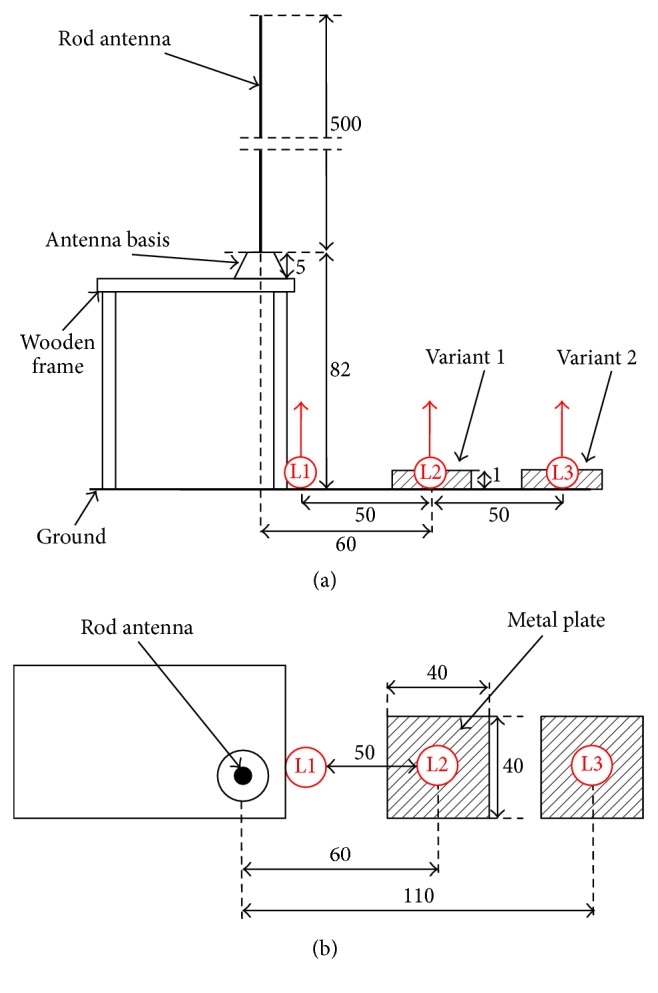
The scheme of exposure set-up used or modelled during* in situ* and* in silico* measurements: (a) side view and (b) top view; L1–L3: locations of measurement lines; dimensions in cm.

The EMR source used in the experimental investigations was a radio communication unit (radiophone) equipped with an upright rod antenna 500 cm long, placed outdoor in the open space at the top of the wooden frame, at a height of 82 cm from the ground (Figure 1), working at a frequency of 27 MHz. Such devices are used for wireless communication and may be mounted on vehicles.

### 2.2. Volunteers

Volunteers, 24 males, with the anthropometric properties presented in Table 1, were involved in the experimental part of the study. They were asked to report anonymously the following properties: height, body mass, chest, and waist circumferences. Additionally, the body mass index (BMI) and the body volume index (BV) values were calculated (Table 1).

### 2.3. *In Situ* Measurements

During the LIC measurements, the volunteers were in a upright posture with lowered upper limbs. They were standing on an aluminium plate. The plate with dimensions of 40 × 40 × 1 cm laid on the ground and its centre was 60 cm (variant 1) or 110 cm (variant 2) apart from the antenna axis (Figure 1). The main axis of the volunteers participating in the research was also fitted in the centre of the plate. The grounding conditions of the volunteers were unified with the use of the plate. In both exposure variants, the following subvariants were investigated: the volunteers used unified shoes (the same, 1a and 2a); they used their own shoes (regular shoes of each volunteer, different, 1b and 2b); and they were barefooted (1c and 2c).

The research protocol concerning the volunteers was approved by the local ethics committee. The clamp-on probe was placed over the clothing (not in direct contact with the body) and the volunteer did not touch any elements of the EMR source, nor any metal object nearby the EMR source. Exposure to EMR was also controlled and did not exceed the worker's exposure limit, being at the typical level of exposure of personnel present near a radiophone in regular use. The volunteers were informed about the aim of the experiment and the principles of the measurement method. They could withdraw at any phase of the research.

In the process of numerical model validation, the *E*-field strength spatial distribution was evaluated. Safety guidelines provide limits to evaluate both *E*- and *H*-field exposure of workers, but it is known from other studies that when evaluating LIC in the lower legs of a worker who stays some distance away from the EMR source, the *E*-field component plays a dominant role [18, 19]. In accordance with D2013/35/EU methodology, spatial distributions of rms (root-mean-square) values in an unperturbed *E*-field (in the absence of workers) strength were evaluated by the* in situ* measurements. *E*-field was measured along three vertical measurement lines: L1, at a distance of 10 cm from the antenna; L2, at a distance of 60 cm (covering the location of the main axis of the body of the volunteers participating in LIC measurements in variant 1, above the centre of the metal plate); and L3, at a distance of 110 cm (covering the location of the main axis of the body of the volunteers participating in LIC measurements in variant 2, above the centre of the metal plate) (Figure 1). The *E*-field strength values were measured along lines L1–L3, at the height above the ground ranging from 30 cm up to 200 cm, with 10 cm steps between successive measurement points.

**Figure 2:**
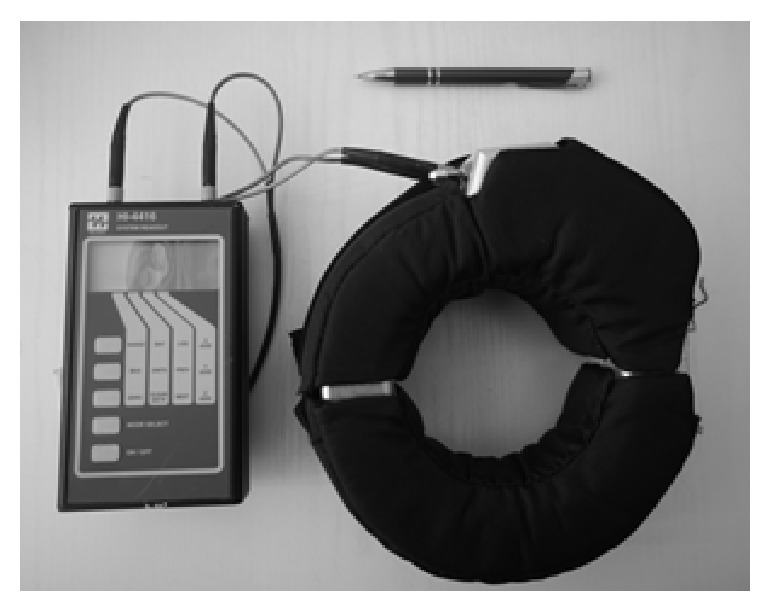
The induction clamp-on meter of limb current (HI-3702 probe and HI-4416 monitor).

The lower limb current measurements at the ankle of volunteers (staying close to the rod antenna) were carried out with the use of an induction (clamp-on) meter HI-3702&HI-4416, manufactured by Holladay, USA (Figure 2) (http://www.ets-lindgren.com), which measures the rms value of current in the range: (0.01–1000) mA; 9 kHz–110 MHz; standard uncertainty of up to ±15%. The research used also a EMR-300 meter manufactured by Wandel & Goltermann, Germany, equipped with an isotropic *E*-field probe of measurement range of rms values: (0.4–800) V/m; 100 kHz–3 GHz, standard uncertainty of up to ±15%.

### 2.4. *In Silico* Measurements

International guidelines and standards require that numerical calculations aimed at evaluating EMR exposure have to reflect the exposure scenario considered at the workplace but do not specify in detail the procedures for such an evaluation [5, 10, 13, 14]. The numerical modelling (*in silico* measurements) reported in this paper covered the following:

- The source of spatially heterogeneous EMR, which mimics rod antenna emitting EMR at a frequency of 27 MHz, is modelled.
- Four* free standing *(nongrounded) numerical phantoms in standing upright posture with lowered upper limbs are used: two models of the female body, Donna and Laura, and two models of the male body, Gustav and Hugo. The dielectric and anthropometric properties of these phantoms were already presented and analysed in the context of correlation with parameters representing the population of adults in various countries [11, 15, 16].
- The results of the* in silico* measurements were validated by the relevant* in situ* measurement results: spatial distribution of *E*-field along lines L1, L2, and L3 and LIC in volunteers.

Numerical calculations were performed with specialized software for EMR analysis, CST Studio [20], based on the finite integration technique (FIT), using Microwave modules* Transient* (time domain solver), boundary conditions* open* (add space), and 15–18 million voxels per numerical model, with smallest voxel in a model of the body 1-2 mm in size. The estimated standard uncertainty of the numerical simulation results of *E*-field distribution near its source is evaluated to be ±(15–18)%. In further analysis, it has been assumed that the uncertainty of numerical simulation of *E*-field distribution is ±18%. The standard uncertainty of* in silico* measurements on LIC (*I*_L_) during exposure to an EMR of 27 MHz matches ±(16–21)%, as estimated based on data in [21, 22]. In further analysis, it has been assumed that the uncertainty of numerical simulation of LIC is ±21%.

### 2.5. Validation of Models

For the purpose of validating the results of numerical calculations, the unperturbed *E*-field strength in the vicinity of rod antenna (affecting the volunteer involved later in the LIC measurements) was analysed with respect to the values of the measured limbs electric current (LIC at ankle) of the volunteers staying there. The* in silico* results were validated by the* in situ* results, using analytical formula to test the identity of the results of two independent studies [23], derived from the assumption that two results obtained with a particular uncertainties are equal (*X*_M_ = *X*_C_):

(1) $$\begin{array}{c} ID=\frac{X_{M}-X_{C}}{\sqrt{U_{M}^{2}+U_{C}^{2}}}, \end{array}$$

where *X*_M_ is *E*-field strength (*E*_M_) or LIC (*I*_LM_) values obtained from measurements, *X*_C_ is relevant *E*_C_ or *I*_LC_ values obtained from numerical calculations, *U*_M_ is standard uncertainty of measurements (±0,15*X*_M_, in the case of *E* or *I*_L_ values), and *U*_C_ is standard uncertainty of numerical calculations (±0,18*X*_C_, in the case of *E* values; ±0,21*X*_C_, in case of *I*_L_ values).

The* in silico* and* in situ* results were taken to be identical when the absolute value of the ID parameter did not exceed unity:

(2) $$\begin{array}{c} \left|\;ID\;\right|\leq 1. \end{array}$$

### 2.6. Statistical Analysis

In the analysis of the correlation between the LIC and (1) the *E*-field strength and (2) the anthropometric properties of the human body, the STATISTICA software version 9.0 PL (StatSoft, USA) was applied. The analysed anthropometric properties of numerical phantoms and volunteers were height, body mass, body mass index (BMI), body volume index (BV), and chest and waist circumferences. Correlation analysis between particular subsets of results was made based on the *r-Pearson *correlation coefficient (when normal distributions of analysed data sets have been proven by a Shapiro-Wilk test (*p* < 0.001)) [23, 24]. The power of correlation was assessed applying the four-step criterion for the *r*-values [25].

## 3. Results and Discussion

The modelled source of heterogeneous EMR (the rod antenna, emitting EMR of 27 MHz frequency) may be treated as the part of scenarios of the exposure representative for diverse situations appearing under real-life conditions, both using military equipment (e.g., the exposure of soldiers staying outside a vehicle equipped with the radiophone)and using civil radio devices (e.g., the exposure of people staying outside the vehicle equipped with citizens band (CB) radio).

**Figure 3:**
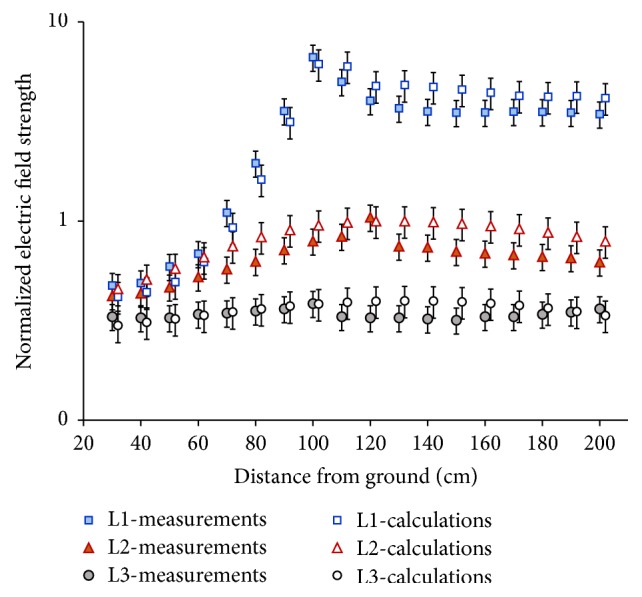
Unperturbed electric field strength in the vicinity of rod antenna of the radiophone: comparison of measured and numerically calculated values along lines L1–L3 (see Figure 1) with standard uncertainty of their evaluation (reference value, maximum value along the line L2).

Figure 3 shows the values of the *E*-field strength, the results of both measurements, and numerical calculations, along with standard uncertainties of their determination, normalized with regard to the maximum value along line L2 (i.e., the axis of exposed body in variant 1).

The distribution of *E*-field level along lines L1–L3 is varying significantly, over 10 times. Therefore, it is not justified to assume that the quasi-homogenous *E*-field exposure was investigated, which was considered in previous studies focused on LIC at lower limbs [19, 25].

**Figure 4:**
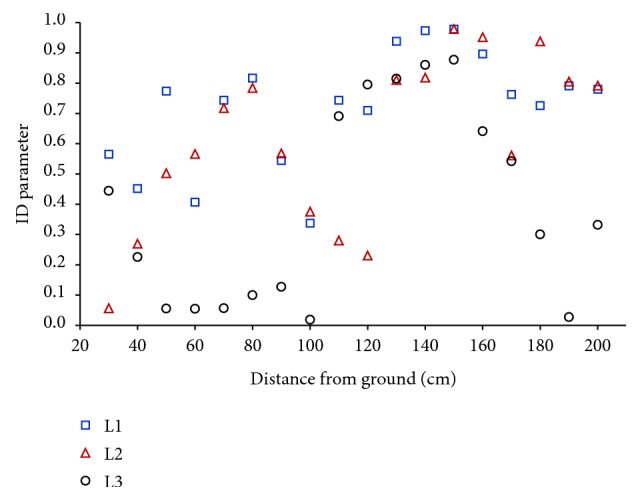
The level of identity (ID parameter, formula (1)) of the results of* in situ* and* in silico* measurements of the unperturbed electric field strength distribution in the vicinity of real rod antenna and its corresponding virtual model; L1–L3 lines (see Figure 1).

An analysis of the spatial distributions of *E*-field strength, obtained from the measurement and numerical calculations, in the vicinity of real rod antenna and its corresponding numerical model, carried out with the use of ID parameter (formula (1)), showed their identity at each of the 90 evaluation points (|ID| ≤ 1) (Figure 4), at the level of mentioned standard uncertainty of their evaluation.

Statistical analysis of the distribution in the analysed results of (1) LIC measurements at the volunteers exposed to the EMF near the radio communication rod antenna and (2) the anthropometric properties of the volunteers, carried out using the Shapiro-Wilk test (*p* < 0.001), showed the normal distribution of all data sets. A correlation analysis showed strong, statistically significant correlations (*Pearson *|*r*| > 0.7, *p* < 0.001) between anthropometric properties of the volunteers: body mass, BV, BMI, and waist and chest circumferences (Table 2).

In both investigated variants of exposure scenario (i.e., two distances from the EMF source), positive, statistically significant correlation was found between LIC measured at the volunteers (*N* = 24) and the volunteers' height or BV index (Table 3). The lowest LIC at barefooted volunteers in variant 1 (*I*_L_ = 29.8 mA) have been found at subject of 172 cm height, 62 kg mass, and 106.6 BV index, when the highest (*I*_L_ = 45.1 mA) have been found at subject of 182 cm height, 120 kg mass, and 218 BV index. The lowest LIC at barefooted volunteers in variant 2 (*I*_L_ = 13.4 mA) have been found at subject of 172 cm height, 62 kg mass, and 106.6 BV index, when the highest (*I*_L_ = 27.8 mA) have been found at subject of 185 cm height, 120 kg mass, and 222 BV index. LIC measured at barefooted volunteers (subvariants 1c and 2c) were 20–40% higher than at volunteers using their own shoes (subvariants 1b and 2b) and 40–50% higher than at volunteers using unified shoes (subvariants 1a and 2a). It suggests that in the exposure scenario near a rod antenna in upright position the regular shoes protect only up to 2 times against LIC; for better protection the soles should be made out of more insulating materials.

**Figure 5:**
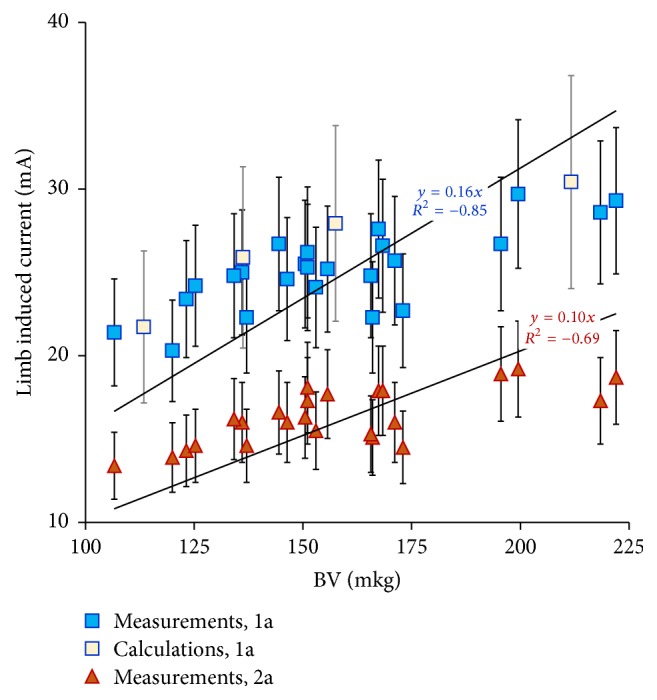
Limb induced current values measured at the volunteers and numerically calculated in the virtual human body phantoms, exposed to EMR emitted by the radio communication antenna (whiskers, standard uncertainty of limb induced current evaluation) in the function of the BV index; variant 1a: volunteers present 60 cm from the antenna; variant 2a: volunteers present 110 cm from the antenna; unified shoes.

Distributions of the values of LIC measured at the volunteers and numerically calculated in the numerical phantoms of the human body exposed to EMF in the vicinity of the radio communication rod antenna (including the standard uncertainty of their evaluation) were shown in Figure 5 in the function of BV index, the anthropometric property found as the strongest correlated with the LIC values.

**Figure 6:**
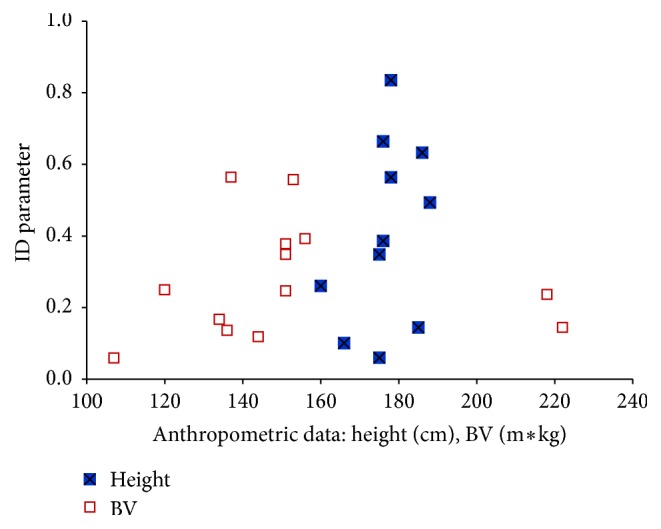
The identity level of limb induced current measured at volunteers and numerically calculated in virtual human body phantoms with the most approximated values of anthropometric properties, exposed to electromagnetic field from radiophone rod antenna: ID parameter (formula (1)).

The LIC measured at the lower limbs (at ankle) of the volunteers were also analysed in order to validate relevant results of numerical calculations. Identity analysis of LIC values measured in the volunteers (*N* = 24) and numerically calculated in phantoms (*N* = 4) was carried out with regard to the subjects of the most approximated values of anthropometric properties. The parameter ID (formula (1)) showed their identity at each investigated case of height and BV index (|ID| ≤ 1), at the level of mentioned standard uncertainties of their evaluation (Figure 6).

The results of LIC measurements at the volunteers were correlated with the following parameters derived from the calculated distribution of absolute value (module) of unperturbed *E*-field (without human body presence), to represent interperson variability of exposure level in the heterogeneous EMR near the rod antenna:

- The *E*-field at the top of the volunteer's body (at the distance from the ground equal to the volunteer's height), along the measurement line L2 or L3, respectively, for exposure variant 1 or 2 (as shown in Figure 1, the line covering the main axis of the volunteer's torso; as shown in Table 1, at the height from the ground in the range 160–190 cm), *E*(BA-TV-L2/L3)
- The arithmetic averaged value of the *E*-field along the main axis of the volunteer's body, respectively, for exposure variant 1 or 2 (line L2 or L3), ranging between 30 cm from the ground and volunteer's height, *E*(BA-AV-L2/L3)
- The *E*-field, located in front of the volunteer's chest (Variant 1 or 2), that is, in line L1 or L2, at a distance from the ground of approximately 70% of the volunteer's height (50 cm from the body axis and, as shown in Table 1, at a distance from the ground in the range 112–133 cm), *E*(FB-0.7VH-L1/L2)
- The maximum in space value of *E*-field affecting the workers' body in exposure variant 1 or 2, according to Directive 2013/35/UE, *E*(D2013/35/EU).

In all investigated exposure variants and their subvariants, the strongest, proportional (physically correct), statistically significant correlations (|*r*| > 0.7, *p* < 0.001) were found between the results of the LIC measurements and the following parameters of EMR exposure (Table 4):

- The maximum in space value of *E*-field affecting the workers' body, *E*(D2013/35/EU)
- The *E*-field in front of the volunteer's chest, *E*(FB-0.7VH-L1/L2).

Both parameters (exposure measures) are more strongly correlated with the LIC values than parameters discussed in the literature [17, 23]: the *E*-field at the top of the volunteer's body *E*(BA-TV-L2/L3) and the arithmetic averaged value of the *E*-field along the main axis of the volunteer's body *E*(BA-AV-L2/L3).

**Figure 7:**
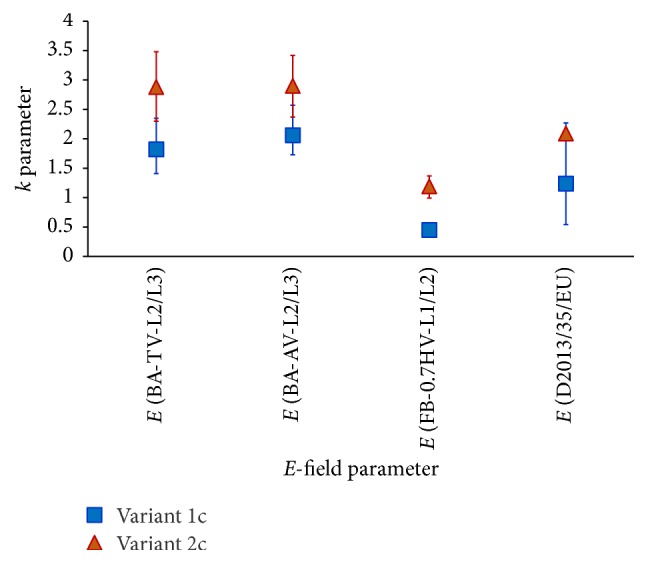
The ratio, *k* = *I*_L_/*E*, between limb induced currents measured at volunteers (*N* = 24) and parameters characterizing the level of *E*-field exposure; arithmetic mean and min–max range (whiskers) in the worst case scenario of exposure (volunteers barefooted); *E*-field parameters are defined in Table 4.

The obtained results indicate that, at the investigated type of workplace EMR exposure, the *I*_L_ values can be evaluated on the basis of mentioned *E*-field parameters shown to be strongly correlated with LIC (Table 4). The lowest uncertainty of such evaluation is provided by the use of *E*-field parameter which creates the values of *k* = *I*_L_/*E* ratio which is the lowest dependent on exposure conditions, *E*-field spatial distribution, and anthropometric properties of exposed workers. In the 6 analysed exposure variants the most stable values of *k*-ratio were found for *E*(D2013/35/EU) and *E*(FB-0.7HV-L1/L2) exposure parameters (Figure 7).

Between the *E*-field exposure measures, which have been shown to be correlated with the LIC, the most practical in the workplace exposure evaluation seems to be *E*(FB-0.7VH-L1/L2), because it needs only a single spot measurement in the location which is easy to be found at the workplace. The main limitation in the use of this measure is exposure at very short distance of worker from the EMR source (shorter than 50 cm). But it needs to be pointed out that, following the ICNIRP's guidelines (referenced by D2013/35/EU), in such a situation direct evaluation of thermal effects is advised, using SAR numerical calculations. In longer distance the evaluation of *E*(FB-0.7VH-L1/L2) may be advised.

The second conclusion may be drawn where in the legal implementation of D2013/35/EU it is necessary to define dimensions of the “reference EMR probe” applicable in the exposure evaluation at the workplace. The Directive statement to use “calculated or measured maximum field” affecting worker's body is significantly unprecise, which may create significant uncertainty in practice. In the vicinity of EMF source some discrepancy between calculated and measured maximum value exists and it depends on the spatial distribution of the exposure level. It is because, during the measurement, EMR is averaged over the volume of the probe. Assuming the 3-dimensional volume of the *E*-field probe to be 10 × 10 × 10 cm (as may be found in the equipment), discrepancy between calculated and measured maximum *E*-field value may be evaluated by the parameter defined by the formula:

(3) $$\begin{array}{c} K_{CM}\left(\;l\;\right)=\frac{E_{C}\left(l\right)-E_{M}\left(l\right)}{E_{M}\left(l\right)}\cdot 100\%, \end{array}$$

where *E*_C_ is calculated maximum value of *E*-field affecting worker (at worker's body position), *E*_M_ is measured maximum value of *E*-field affecting worker, estimated based on the calculated value of *E*-field averaged over the volume of the probe (assumed to be of 10 × 10 × 10 cm dimensions), and *l* is location under consideration (where max *E*-field was found).

In the vicinity of rod antenna the following discrepancy (*K*_CM_) was found: 190% at the distance of 2 cm from the antenna, 80% at 5 cm, 40% at 10 cm, 9% at 60 cm, and 7% at 110 cm. It is shown that the discrepancy between measured and calculated maximum value of *E*-field, which is affecting worker, may significantly contribute to the uncertainty of exposure evaluation (and evaluation of the compliance with exposure limits). In small distance from the EMR source, mentioned influence is stronger than from the measurement or calculations uncertainty itself. In the distance from the rod antenna exceeding 60 cm, the analysed discrepancy is less than 10% (being within the range of standard uncertainty of *E*-field measurements itself).

The other technical limitation in evaluating EMR exposure near the source comes from the properties of EMR measurement devices, which may be directly coupled with the EMF source and indicates not calibrated values. In consequences, the measure of EMR exposure, which is the strongest correlated with hazards related to the LIC in the radiofrequency EMR, may be not achievable by measurements in the work environment.

In volunteers of the height 160–190 cm exposed to EMR from radiophone rod antenna at the level of *E*-field strength equal to 61 V/m (established by D2013/35/EU as the maximum value of *E*-field strength in the workers' body position, in the absence of worker), the LIC (at ankle) ranged from 45% up to 85% in variant 1 of exposure scenario and from 76% up to 144% in variant 2 of its limit values, provided by D2013/35/EU (100 mA). Limb induced current in the worst case of exposure, barefooted workers on grounded basis (variants 1c and 2c), evaluated based on the *k* = *I*_L_/*E* ratio exceeds 50% of mentioned limit (*I*_L_ > 50 mA) at the level of exposure to *E*-field expressed as *E*(D2013/35/EU) > 20 V/m or *E*(FB-0.7HV-L1/L2) > 35 V/m. Both exposure levels are below the directive limits of* E*-field strength in the workplace. Because of the negative correlation between LIC and exposed person height values, in analysed exposure conditions, the highest volunteer does not represent the worst case of LIC value at the particular workplace.

Results presented in this paper showed that in the distance from the antenna exceeding 100 cm (variant 2 of exposure scenario) the compliance with *E*-field exposure limits does not ensure automatic compliance with LIC limits. Taking into consideration limited availability of LIC measurement devices, in the workplace under the questionable exposure condition, the application of sufficient protection measures should be advised. In that context it needs attention that, in the investigated exposure cases near the upright rod antenna, it was found that regular shoes insufficiently protect workers; they are reducing the LIC values only up to 2 times. In the case of the need of better protection against EMR exposure effects it is necessary to use shoes equipped with the soles made from materials which are insulators for radiofrequency currents.

## 4. Conclusions

*In situ* and* in silico* measurements of limb induced current at ankle of volunteers of height in the range 160–190 cm exposed to electromagnetic field of 27 MHz frequency, emitted from nearby radiophone upright rod antenna 500 cm long (e.g., such as that used in military vehicles), showed in all exposure scenarios the strong, statistically significant correlation between limb induced current and the body volume index of exposed persons (body mass multiplied by body height, in kg*∗*m) (*r* > 0.7; *p* < 0.001).

The relations between the limb induced current and selected parameters of unperturbed electric field affecting the workers' body are allowing for estimating the range of values of lower limbs currents experienced by exposed persons. The most informative exposure parameters, with respect to the evaluation of electromagnetic hazards by measurements (the strongest correlated with limb induced current), were found to be the value of electric field in front of chest (50 cm from body axis) or the maximum value in space occupied by human (unperturbed field exists in the investigated space in the absent of human body).

Exposed person's body volume and electric field strength in front of the chest determine limb induced current in studied exposure scenarios but mentioned parameters have not been analysed yet in other studies and they need further studies regarding their usefulness in other exposure scenarios in the workplace.

## Figures and Tables

**Table 1:** Statistical parameters of anthropometric properties reported by volunteers.

Statistical parameter	Anthropometric properties (*N* = 24)					
	Height	Body mass	BV	BMI	WC	CC
	(cm)	(kg)	(m*∗*kg)	(kg/m^2^)	(cm)	(cm)
Mean ± SD	178.2 ± 7.1	88.2 ± 14.7	157 ± 29	27.8 ± 4.3	101.1 ± 11.3	106.8 ± 9.7
Median	179.5	86.0	152	27.5	99	105
IQR	172.8–182.3	77.8–94.8	136–168	24.9–29.3	95.8–105.8	101.8–108.5
Min–Max	160–190	60–120	106–222	21.0–36.3	82–129	93–135

SD: standard deviation; IQR: interquartile range (range between 25. and 75. percentile values); body mass index: BMI = body mass/(height)^2^; body volume index: BV = height *∗* body mass. WC = waist circumference; CC = chest circumference.

**Table 2:** The correlation between anthropometric properties of volunteers (*N* = 24).

	Height		Body mass		BV		BMI		WC		CC	
	*r*	*p*	*r*	*p*	*r*	*p*	*r*	*p*	*r*	*p*	*r*	*p*
Height	1.0	—	0.40	0.06	0.58	0.003	−0.11	0.62	0.06	0.80	0.11	0.62
Body mass	0.40	0.06	1.0	—	**0.98**	**<0.001**	**0.87**	**<0.001**	**0.89**	**<0.001**	**0.88**	**<0.001**
BV	0.58	0.003	**0.98**	**<0.001**	1.0	—	**0.74**	**<0.001**	**0.77**	**<0.001**	**0.81**	**<0.001**
BMI	−0.11	0.62	**0.87**	**<0.001**	**0.74**	**<0.001**	1.0	—	**0.90**	**<0.001**	**0.89**	**<0.001**
WC	0.06	0.80	**0.89**	**<0.001**	**0.77**	**<0.001**	**0.90**	**<0.001**	1.0	—	**0.85**	**<0.001**
CC	0.11	0.62	**0.88**	**<0.001**	**0.81**	**<0.001**	**0.89**	**<0.001**	**0.85**	**<0.001**	1.0	—

Strong, statistically significant correlations (|*r*| > 0.7, *p* < 0.001) are in bold; body mass index: BMI = body mass/(height)^2^; body volume index: BV = height *∗* body mass; WC = waist circumference; CC = chest circumference; *r-Pearson *correlation coefficient and significance level *p*.

**Table 3:** The correlation between the limb induced current measured at volunteers (*N* = 24) exposed to the electromagnetic field (27 MHz) near the rod antenna and the volunteers' anthropometric properties.

Anthropometric properties	Exposure variants											
	1a		1b		1c		2a		2b		2c	
	*r*	*p*	*r*	*p*	*r*	*p*	*r*	*p*	*r*	*p*	*r*	*p*
Height	**0.74**	**<0.001**	0.65	0.001	0.49	0.014	**0.70**	**<0.001**	0.63	0.001	0.64	0.001
Body mass	0.67	<0.001	**0.74**	**<0.001**	**0.82**	**<0.001**	0.63	<0.001	**0.74**	**<0.001**	**0.75**	**<0.001**
BV	**0.76**	**<0.001**	**0.80**	**<0.001**	**0.84**	**<0.001**	**0.71**	**<0.001**	**0.80**	**<0.001**	**0.81**	**<0.001**
BMI	0.31	0.147	0.46	0.025	0.60	0.002	0.29	0.165	0.46	0.022	0.46	0.022
WC	0.32	0.128	0.47	0.020	0.59	0.002	0.27	0.197	0.44	0.033	0.50	0.012
CC	0.39	0.059	0.50	0.014	0.65	0.001	0.37	0.076	0.49	0.016	0.54	0.007

Body mass index: BMI = body mass/(height)^2^; body volume index: BV = height *∗* body mass; WC = waist circumference; CC = chest circumference; variant 1 of exposure: the axe of volunteer's body 60 cm away from the antenna; variant 2 of exposure: the axe of volunteer's body 110 cm away from the antenna; a: the volunteers used unified shoes; b: the volunteers used their own (regular) shoes; c: the volunteers were barefooted; strong, statistically significant correlations (|*r*| > 0.7, *p* < 0.001) are in bold; *r*-*Pearson *correlation coefficients and significance level *p*.

**Table 4:** The correlation between the electric field parameters and the limb induced current measured at volunteers (*N* = 24) exposed to the electromagnetic field (27 MHz) near the rod antenna (all measurements: 1a, 1b, 1c, 2a, 2b, 2c; *N* = 144 cases).

*E*-field parameter	Limb induced current measured during exposure near the rod antenna	
	*r*	*p*
*E*(BA-TV-L2/L3)	**0.724**	**<0.001**
*E*(BA-AV-L2/L3)	**0.746**	**<0.001**
*E*(FB-0.7HV-L1/L2)	**0.747**	**<0.001**
*E*(D2013/35/EU)	**0.770**	**<0.001**

Strong, statistically significant correlations (|*r*| > 0.7, *p* < 0.001) are in bold; the parameters of the calculated unperturbed *E*-field related to the absolute (ABS) value of the *E*-field root-mean-square (RMS) value: *E*(BA-TV-L2/L3): the *E*-field calculated at the top of the volunteer body, along the measurement line L2 or L3, respectively, for exposure variant 1 or 2 (as shown at Figure 1, the line covering the main axis of the volunteer's torso); *E*(BA-AV-L2/L3): the arithmetic average value of the *E*-field calculated along the main axis of the volunteer's body, line L2 or L3, respectively, for exposure variant 1 or 2, ranging between 30 cm from the ground and volunteer's body height; *E*(FB-0.7VH-L1/L2): the *E*-field calculated in line L1 or L2, located in front of the volunteer's chest (variant 1 or 2); *E*(D2013/35/EU): the maximum in space of *E*-field value of field affecting the workers' body in exposure variant 1 or 2, according to Directive 2103/35/EU; *r-Pearson* correlation coefficients and significance level *p*.
